# Molecular identification of Palearctic members of *Anopheles maculipennis *in northern Iran

**DOI:** 10.1186/1475-2875-6-6

**Published:** 2007-01-17

**Authors:** Navid D Djadid, Saber Gholizadeh, Elham Tafsiri, Roberto Romi, Mikhail Gordeev, Sedigheh Zakeri

**Affiliations:** 1 Malaria Research Group, Biotechnology Department, Institut Pasteur of Iran, Tehran, Iran; 2 Laboratorio di Parassitologia, Instituto Superiore di Sanita, Rome, Italy; 3 Vavilov Institute of General Genetics, Russian Academy of Sciences, Moscow, Russia

## Abstract

**Background:**

Members of *Anopheles maculipennis *complex are effective malaria vectors in Europe and the Caspian Sea region in northern Iran, where malaria has been re-introduced since 1994. The current study has been designed in order to provide further evidence on the status of species composition and to identify more accurately the members of the maculipennis complex in northern Iran.

**Methods:**

The second internal transcribed spacer of ribosomal DNA (rDNA-ITS2) was sequenced in 28 out of 235 specimens that were collected in the five provinces of East Azerbayjan, Ardebil, Guilan, Mazandaran and Khorassan in Iran.

**Results:**

The length of the ITS2 ranged from 283 to 302 bp with a GC content of 49.33 – 54.76%. No intra-specific variations were observed. Construction of phylogenetic tree based on the ITS2 sequence revealed that the six Iranian members of the *maculipennis *complex could be easily clustered into three groups: the *An. atroparvus – Anopheles labranchiae *group; the paraphyletic group of *An. maculipennis, An. messeae, An. persiensis; *and *An. sacharovi *as the third group.

**Conclusion:**

Detection of three species of the *An. maculipennis *complex including *An. atroparvus, An. messae *and *An. labranchiae*, as shown as new records in northern Iran, is somehow alarming. A better understanding of the epidemiology of malaria on both sides of the Caspian Sea may be provided by applying the molecular techniques to the correct identification of species complexes, to the detection of Plasmodium composition in Anopheles vectors and to the status of insecticide resistance by looking to related genes.

## Background

*Anopheles maculipennis*, the historic vector of malaria in Europe and the Middle East was the first sibling species complex to be discovered among mosquitoes [1,2]. The Maculipennis complex formally comprised 12 Palearctic members including *Anopheles atroparvus*, *Anopheles beklemishevi*, *Anopheles labranchiae*, *Anopheles maculipennis*, *Anopheles martinus*, *Anopheles melanoon*, *Anopheles messeae*, *Anopheles sacharovi*, *Anopheles persiensis*, *An. daciae*, *An. lewisi *and *An. artemievi *[3-7]. *Anopheles sacharovi *is the main vector in Turkey and is, together with *Anopheles superpictus *and *Anopheles pulcherrimus*, the most important vector, of malaria in the former Soviet Union, although *An. messeae *has been implicated in the resurgence of malaria in Russia and the Ukraine [8]. Three species of the Maculipennis complex, *An. atroparvus*, *An. labranchiae *and *An. sacharovi *are known to be efficient current or historical vectors of malaria in the Palearctic region [9,10]. *An. maculipennis *s. s. has been identified as the major vector of malaria on the Caspian Sea coast area of Iran and *An. sacharovi *is considered the main vector in the central plateau of the country [11,12]. Djadid [13] reported that members of this species complex in northern Iran are active from May to September, with a peak in July and that they breed readily in rice fields, spring and clean standing water, and adult mosquitoes could be found in animal shelters (95%). These species were susceptible to dieldrin, malathion, deltamethrin and resistant to DDT. Their biting pattern on human and animals baits is more or less the same, starting at 19.00 hrs with a peak between 20.00 – 21.00 hrs. Enzyme-linked immunosorbent assay (ELISA) carried out on 304 blood-fed mosquitoes collected from indoor resting sites revealed that they have fed predominantly on cattle, with fewer blood meals on sheep and poultry [13]. None of the mosquitoes had fed on human blood although a previous study by Manouchehri *et al*. [14] has shown an anthropophilic index for this species in northern Iran of 1.7–4.9%. Furthermore, Djadid [13] reported that hibernation in this species starts in October and that complete fat body could be seen in February. The following culicidae mosquitoes larvae have been found in *An. maculipennis *breeding places; *Anopheles hyrcanus, Anopheles claviger, Culex pipiens, Culex mimeticus, Culex tritaeniorhynchus, Aedes vexans, Culiseta subochra, Uranotaenia unguiculata*. Adult of *Anopheles algeriensis *and *Anopheles hyrcanus *also has been found in resting places of *An. maculipennis *[13].

From an operational point of view in malaria control, in the region to the north of the Zargors range of mountains, *An. maculipennis*, *An. sacharivi *and *An. superpictus *are recognized as malaria vectors. This region was malaria-free for more than 30 years. However, since 1994, malaria has been re-introduced to this area through Republic of Azerbaijan and Armenia [15,16].

Several studies have employed ecological, morphological, physiological and biochemical data to characterize members of the *An. maculipennis *complex including the preferences [17], larval chaetotaxy [18], ovarian polytene chromosome banding pattern [19,20] cuticular hydrocarbons [21] isoenzyme analysis [22,23] and most recently, DNA sequences [24-30]. Of these methods, egg morphology [31] and DNA sequencing [7,32,33] have been used for identification of different members of this complex in Iran.

Few studies have been carried out in northern areas of the country where malaria has reappeared and been introduced into several different provinces. Although, it has been postulated that members of *An. maculipennis *complex are responsible for malaria transmission, how many species within maculipennis complex are present in Caspian Sea region has not been defined yet. Do they exist as sympathric species and what if any, is, their role in malaria transmission? In view of the zoogeographical, ecological and social changes on both sides of Caspian Sea, this study was carried out in order to provide further evidence on the status of species composition and to identify more accurately the members of *An. maculipennis *complex in northern Iran. Field collection, morphological identification followed by amplification and sequencing of ITS2 region led to identification of six members of *An. maculipennis *complex and their comparison with those related sequences deposited in GenBank. The occurrence of three species of *An. maculipennis *complex including *An. atroparvus, An. messae *and *An. labranchiae *is reported for the first time from northern Iran. This proves the extended distribution of these species towards southern territory of the *An. maculipennis *complex. This is a pre-requisite for understanding the epidemiology of malaria on both sides of Caspian Sea, and attempting to prevent the re-introduction of malaria to malaria-free areas.

## Materials and methods

### Mosquitoes and ecological characteristics of collection sites

**Figure 1:**
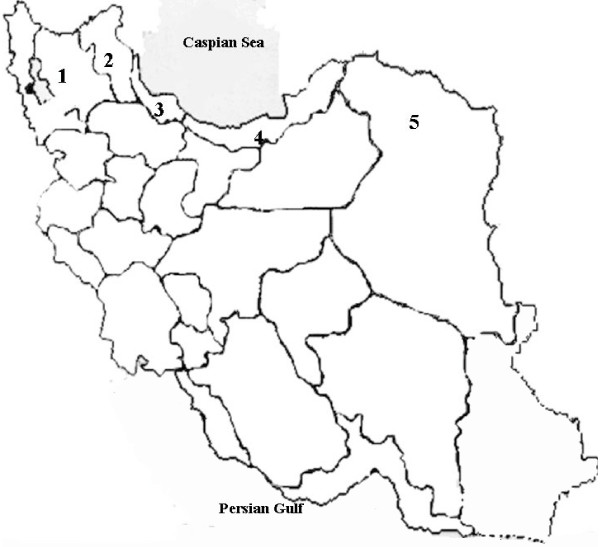
Collection sites of *Anopheles maculipennis *complex specimens in Iran. Numbers quoted in the map (1–5) corresponds to the study areas; East Azerbaijan (1), Ardebil (2), Guilan (3), Mazandran (4) and Khorassan (5) provinces, accordingly.

235 mosquito specimens of the *An. maculipennis *complex were collected by total catch in human and animal shelters in northern Iran including provinces of East Azerbayjan, Ardebil, Guilan, Mazandaran and Khorassan (Figure 1) during three collections in May 1997 to September 1999 and May to September 2001.

**Figure 2:**
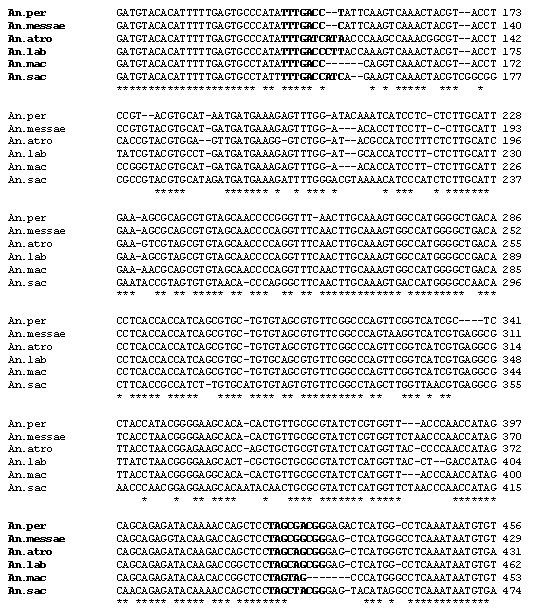
Alignment showing the inter-specific variability in ITS2 sequence for six Palearctic members of *An. maculipennis *complex from northern Iran. The first 26 bp belongs to 5.8 s region and ITS2 starts and ends with bold nucleotides. The remainder 27 bp is 28 s region. Selected sequences used for this alignment could be reached through GenBank accession numbers; AY730269 (*An. persiensis*), AY050639 (*An. messaea*), AY050640 (*An. atroparvus*), AY842516 (*An. labranchiae*), AY730264 (*An. maculipennis*), AY533852 and AY842515 (*An. sacharovi*).

**Figure 3:**
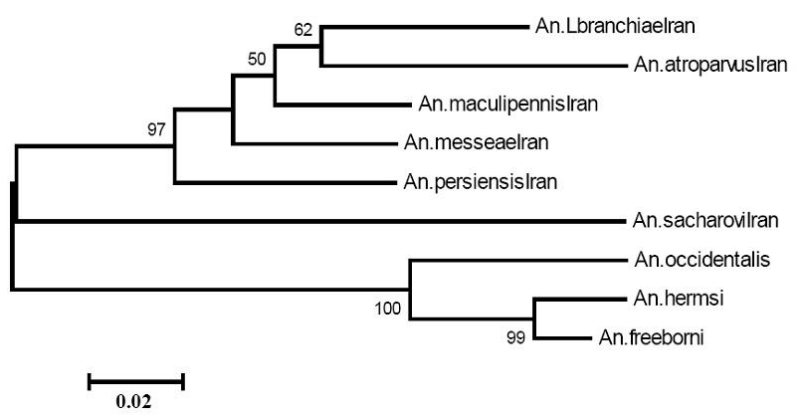
Phylogenetic relationship between Iranian and Nearctic members of *An. maculipennis *complex; *Anopheles hermsi *[GenBank: M64483], *Anopheles freeborni *[GenBank: M64484], *Anopheles occidentalis *[GenBank: M64482], constructed based on rDNA-ITS2 sequence. Bootstrap values under 50% have not shown.

**Figure 4:**
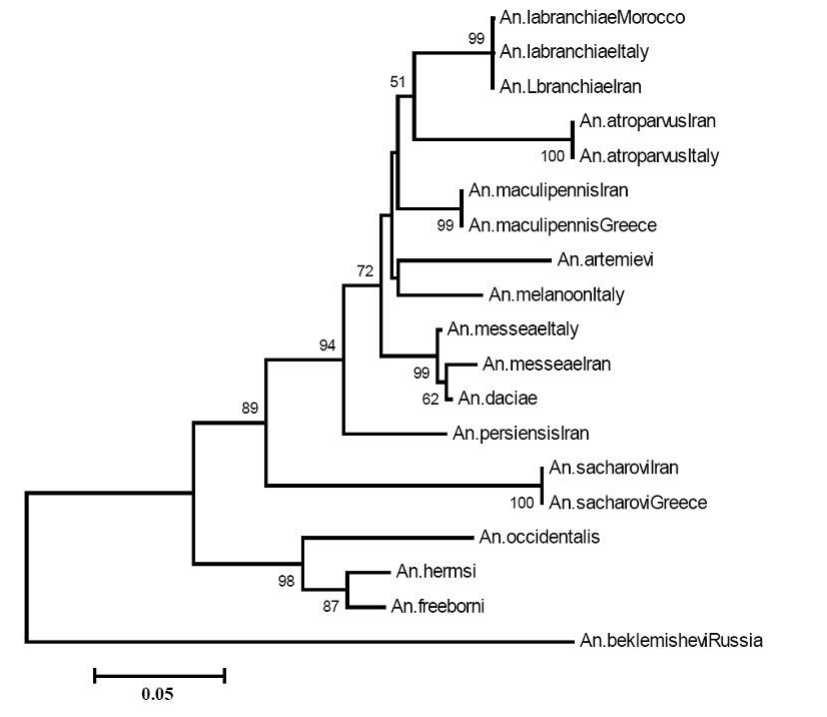
rDNA-ITS2 sequence generated the phylogenetic tree, showing the relationship between members of Maculipenni*s *complex from old and new world. *An. persiensis *[GenBank: AY730269, AY137847, Iran], *An. maculipennis *[GenBank: AY730264, AY137798, Iran; Z50104, Italy], *An. sacharovi *[GenBank: AY842517, AY114204, Iran; Z83198, Italy], *An. labranchiae *[GenBank: AY253849, Morocco; Z50102, Italy; AY842516, Iran], *An. messeae *[GenBank: AY050639, Iran; Z50105, Italy], *An. atroparvus *[GenBank: AY050640, Iran; Z50103, Italy], *An. melanoon *[GenBank: AJ224330, Italy], *An. daciae *[GenBank: AY634502], *An. artemievi *[GenBank: AJ849886], *An. beklemishevi *[GenBank: AY593958], *An. hermsi *[GenBank: M64483], *An. freeborni *[GenBank: M64484] and *An. occidentalis *[GenBank: M64482]. Bootstrap values under 50% have not shown.

Two mountainous provinces of East Azerbaijan and Ardebil sharing border line with republics of Azerbaijan and Armenia, while Guilan and Mazandran with Mediteranean climate are located in southern coast of Caspian Sea. Eastern corner of Mazandran and northern Khorassan are close to republic of Turkmenistan. However, the whole eastern part of Khorassan province is in border of Afghanistan. With respect to malaria transmission, all these five provinces have one peak during June-August. Details regarding the origin and number of specimens used for PCR amplification and sequencing are given in Table 1 and Figures 1, 2, 3, 4.

**Table 1:** Accession numbers assigned for twenty-eight rDNA-ITS2 sequence in six members of *Anopheles maculipennis *complex from five provinces in northern Iran.

**Species**	**Province**	**GenBank Accession number**
***An. maculipennis***	Khorassan	AY730264
	Guilan	AY730265, AY842514, DQ243829, DQ243830
	Mazandaran	AY730267, AY730268
	Ardebil	AY533853
	East Azerbayjan	AF436065
***An. sacharovi***	Ardebil	AY533852, AY842515, AY842517
	Guilan	DQ243825, DQ243826, DQ243827, DQ243828DQ243832, DQ243833
***An. persiensis***	Mazandaran	AY730269
	Guilan	AY730266, AY842519, AY702491, DQ243831, DQ243834
***An. atroparvus***	Mazandaran	AY050640, AF436064
***An. messeae***	Guilan	AY050639
***An. labranchiae***	Guilan	AY842516

### Mosquitoes' morphological identification

On arrival in the laboratory of malaria research group (MRG) in the Institut Pasteur of Iran, mosquitoes were identified by a morphological key of Iranian Anophelines [34] to distinguish *An. maculipennis *and *An. sacharovi *adults from other Anopheline species.

### Mosquito genomic DNA extraction and PCR amplification

Mosquito genomic DNA was extracted using slight modification of the method described by Collins *et al*. [35]. The ITS2 region of rDNA gene was amplified using the universal primers of 5.8 s (5' ATC ACT CGG CTC GTG GAT CG 3') and 28 s (5' ATG CTT AAA TTT AGG GGG TAG TC 3'). All PCR reactions were performed in a total volume of 25 μl. The reaction mixture contained 50 ng of each of the specific primers of 5.8 s and 28 s, which are flanking the whole ITS2 and partial sequence of 5.8 s and 28 s regions at both ends [36], 0.5 unit of Taq polymerase, 0.1 mM each of dNTPs, 0.001% gelatine, 2.5 μl of 10X reaction buffer, 2 mM MgCl_2_. The amplification profile was as follows: denaturation at 94°C for 5 min, followed by 30 cycles of annealing at 53°C for 1 min and extension at 72°C for 1 min with 7 min extra extension time in the last cycle. The target amplified DNA was run on 1.5% agarose gel. Gels were stained with ethidium bromide and bands were visualized by UV transillumination.

### Sequencing of PCR products

Sequencing was performed for selected specimens based on the size of their PCR product, collection site and prevalence of each species. Amplified fragments were purified by QIAquick^® ^(Germany) kit, and subjected to sequencing from both ends in an ABI-373 automatic sequencer by Primm Company (Italy), using the same amplification primers of 5.8 s or 28 s.

### Data analysis

The sequencing signals in *An. maculipennis *specimens were double-checked and annotated followed by comparison with GenBank data and previously published [32,37] and unpublished sequences from the Malaria Research Group (MRG) in the Biotechnology Department at the Institut Pasteur of Iran. Because of the conserved nature of the partial sequence of 5.8 and 28 s, the alignment of sequences and construction of the phylogenetic tree were performed using only the whole ITS2 sequence in Gene Runner (version 3.05, 1994, Hastings Software Inc), ClustalW [38], ClustalX [39] and Molecular Evolutionary Genetic Analysis (MEGA2) [40] programmes.

## Results

The rDNA-ITS2 region was amplified by PCR from genomic DNA of 235 specimens from the five provinces of East Azerbaijan, Ardebil, Guilan, Mazandran and Khorassan in Iran (Figure 1). Sequences for the ITS2 region were obtained from 28 specimens, which have been deposited in GenBank (Table 1). The size of sequenced PCR amplified fragments in different geographical populations of the *An. maculipennis *complex varied from 453 bp in *An. messeae *to 498 bp in *An. sacharovi*, but with no intra-specific variation. Based on comparison of these sequences with the available ITS2 sequences of *An. maculipennis *complex in GenBank, six known species of the complex were identified in Iranian specimens including: *An. maculipennis *(100% identity with GenBank: AY365010 from Greece), *An. sacharovi *(100% identity with GenBank: AY533588 from Greece), *An. persiensis *(100% identity with GenBank: AY137819 from Iran), *An. messeae *(99% identity with GenBank: AY504236 from U.K.), *An. atroparvus *(99% identity with GenBank: AY365007 from Italy) and *An. labranchiae *(100% identity with GenBank: AY365008 from Italy). The sequence alignment of the six Anopheles species is shown in Figure 2. Presumptive boundaries of 5.8 s and 28 s genes were deduced from the comparison of alignments in the sequences of other mosquitoes [26,37,41]. The size of ITS2 sequences in six species ranged from 283 bp in *An. maculipennis *to 302 bp in *An. sacharovi *(Figure 2). The ITS2 region in all six species identified molecularly in this study started with TTGACC except *An. Atroparvus*, which started with TTGA**T**C. These sequences were well conserved and almost identical to those previously reported for Nearctic and Palearctic taxa of *An. maculipennis *complex [7,26,42]. No intra-specific variation was detected in the ITS2 sequences of either species.

The overall average base composition was 24.89% (23.21–28.48%) for A, 22.36% (21.77–23.19%) T, 24.91% (23.43–25.58%0) G and 27.84% (25.83–28.91%) for C. Percentage of GC content was 53.35 in *An. maculipennis *(ITS2 = 283 bp), 49.33 in *An. sacharovi *(ITS2 = 302 bp), 51.74 in *An. persiensis *(ITS2 = 286 bp), 54.76 in *An. atroparvus *(ITS2 = 294 bp), 53.74 in *An. messeae *(ITS2 = 292 bp) and 53.58 in *An. labranchiae *(ITS2 = 293 bp). These values are concordant with 49.4–54.1% GC values reported for other Palearctic members of *An. maculipennis *complex [26,27,43,44].

### Phylogenetic analysis

The presumptive nucleotide sequences of the rDNA-ITS2 region in nine Palearctic members of the *An. maculipennis *species complex generated in this study and by others [7,26,29,30,45,46] and also three Nearctic taxa [42] were examined for phylogenetic analysis.

Estimating of Neighbor Joining for all pairs of the 28 ITS2 sequences and the phylogenetic tree produced using Clustal method have shown that the Iranian members of *An. maculipennis *complex could be easily clustered, including *An. atroparvus/An. labranchiae *group, the paraphyletic group of three species *An. maculipennis, An. messeae, An. persiensis *and *An. sacharovi *as the third group. Including the New World members of *An. maculipennis *(*Anopheles freeborni, Anopheles occidentalis*, and *Anopheles hermsi*) in the phylogenetic tree revealed a separate clade for each Old and New World member, allocating *An. sacharovi *as the most basal and somewhat isolated member of the Old World *An. maculipennnis *complex (Figure 3).

When all 20 different sequences from GenBank were used to generate the phylogenetic tree, it confirmed the systematic relation of those species from different areas in Iran, Italy, Morocco, Greece, Russia and United Kingdom (Figure 4). *Anopheles daciae *is thus more like *An. messeae*, while *An. melanoon *is the closest taxa to *An. artemievi*. On the other hand, it is clear that because of the difference in ITS2 sequence of *An. beklemishevi *as compared to other Palearctic members of this complex (638 bp/~300), the species is distinct from the other Old World members of the *An. maculipennis *complex, and actually is closer to the Nearctic than to the Palearctic *maculipennis *species or at least as an out group of the Palearctic members.

## Discussion

The group of Anopheles mosquitoes referred to as the Maculipennis complex include the most important malaria vector of the Palearctic region, which is difficult or impossible to identify by their morphological characteristics [47]. However, for the identification of two of the Nearctic *An. maculipennis *species, *An. hermsi *and *An. freeborni*, a PCR assay has already been established [42]. Subsequently, Proft *et al*. [27] developed a diagnostic PCR system to differentiate between six of the seven *An. maculipennis *sibling species occurring in Europe including *An. maculipennis, An. sacharovi, An. melanoon*, *An. atroparvus, An. labranchiae *and *An. messeae*. Romi *et al *also designed a heteroduplex analysis based on the ITS2 sequence which enabled seven species of the complex including *An. maculipennis, An. sacharovi, An. martinius*, *An. atroparvus, An. labranchiae, An. melanoon *and *An. messeae *to be identified [47].

This study has been designed in order to provide molecular evidence and to verify the real composition of the *An. maculipennis *complex in northern Iran. The mosquito fauna in Iran has not been extensively studied since 1986 [48] and later when Anophelines larvae were studied by Saebi [49]. However, regarding the *An. maculipennis *species complex, Faghih *et al*. [50] and Manouchehri *et al*. [14] claimed the presence of four species including *An. maculipennis *s.s. (typicus) in Ramsar (Mazandran province), *Anopheles subalpinus *in Sari, Babolsar, Chalous (Mazandran province), Astaneh (Guilan province), *An. melanoon *in Astaneh (Guilan) with *An. subalpinus *and *An. melanoon *sympathric in Astaneh and on the border of the two provinces of Guilan and Mazandran in northern Iran. They reported the presence of *An. sacharovi *in all areas of these two provinces. Djadid [32], working on *An. maculipennis*, *An. sacharovi *from Iran, *An. beklemishevi *and two cytogenetically different forms of *An. messeae *from Russia has found three species of *An. sacharovi *[GenBank: AY842517], *An. messeae *[GenBank: AY050639], and *An. atroparvus *[GenBank: AY050640] in Iran by using RAPD, SSR and ITS2 sequences. Recently, Sedaghat *et al*. [7,51] reported the presence of three genetically distinct species of the *An. maculipennis *complex from Iran, including *An. maculipennis*, *An. sacharovi*, and *An. persiensis*. The sequence of *An. persiensis *was identified in 2002 by Djadid and Romi from Rasht (Guilan province) and Amol city in Mazandran province, and indeed it was described later by Sedaghat *et al*. [7]. This species has been found only in the northern Caspian Sea littoral provinces of Guilan and Mazandran. Oshaghi *et al*. [33], working on members of *An. maculipennis *complex from the north-west and central regions of Iran, only found *An. maculipennis *and *An. sacharovi*. However, in study, for the first time, the presence of six species of this complex (*An. maculipennis, An. sacharovi, An. persiensis*, *An. atroparvus, An. labranchiae *and *An. messeae*) are reported in northern Iran based on the sequence of rDNA-ITS2. The three species of *An. messeae, An. atroparvus *and *An. labranchiae *have not been reported before in Iran.

The ITS2 sequences of six Palearctic species of *An. maculipennis *complex varied in length from 283 bp in *An. maculipennis *up to 302 bp in *An. sacharovi*. This is in the range of ITS2 length in other examined Anopheles species; 363–369 bp in *Anopheles nunestovari *[52], 287–329 bp in *Anopheles quadrimaculatus *complex [53], in the North American species of the *An. maculipennis *complex 305 – 310 bp [42] and in seven Palearctic members of *An. maculipennis *complex about 280–300 bp [26]. However, the sequence of each species from Iran and other parts of the world within this complex is highly conserved, with about 99–100% similarity.

In a previous study by Marinucci *et al*. [26], the phylogenetic relationships among the members of the *An. maculipennis *complex inferred by maximum parsimony analysis of the PAUP programme and neighbour joining and maximum likelihood analysis of the PHYLIP program. All the trees obtained were almost identical in topology although the relationships among the three species i.e. *An. maculipennis, An. messeae *and *An. melanoon*, remained unresolved. Perhaps due to the differentiation of these species from neighbouring taxa within a brief evolutionary time-frame that dispensed insufficient differences to support these individuals' lineages [26]. Recently rDNA-ITS2 sequences of three other Palearctic members of the *An. maculipennis *complex have been reported from Romania and Russia, namely *An. daciae*, *An. artemievi *and *An. beklemishevi *[29,30,45,46]. Kampen analysed *An. beklemishevi *specimens from Russia by their ITS2 ribosomal DNA sequences to amend and to specify the phylogenetic tree of the *An. maculipennis *species complex [30]. The results generally correspond with the data presented in the current study except that the constructed trees do not include *An. persinsis*, and that *An. messeae *and *An. atroparvus *are not as close as demonstrated by Kampen [30]. He showed that *An. beklemishevi *is in a closer relationship to the Nearctic rather than to the Palearctic sibling species, which is in concordance with the demonstration of final phylogenetic tree drawn from this study by including *An. beklemishevi *sequence (GenBank: AY593958) to other sequences (Figure 4). However, *An. sacharovi *as a Palearctic member of *An. maculipennis *complex perhaps due to its common evolutionary speciation, seems to be the closest taxa to both the Nearctic species and *An. beklemishevi*.

The phylogenetic tree generated in the current study has separated Nearctic members of *An. maculipennis *complex into two distinct lineages (Figure 3). In agreement with Marrinucci *et al*. [26], *An. occidentalis *was placed in a sister group in relation to *An. hermsi-An. freeborni*. *An. sacharovi *placed in a sister group to the remainder of the Palearctic group. The *An. labranchiae-An. atroparvus *clade was the sister group to *An. maculipennis*, *An. persiensis *and *An. messeae*. However, interestingly, the newly described member of complex, *An. perciensis *is closer to *An. sacharovi*, perhaps revealing its common evolutionary background with this species as compared to other members of *An. maculipennis *(Figures 3 and 4).

With regards to the presence of six members of *An. maculipennis *complex, further complementary ecological studies are needed in order to determine the role of each species in malaria transmission in different areas of northern Iran. To achieve this goal, some field experiments were carried out. The preliminary results of dissection of *An. sacharovi *(species identification confirmed by ITS2 sequence analysis) collected during the suspected hibernation period (October-March) showed that this species will go into hibernation with gonotrophic dissociation. In this case, all dissected mosquitoes have shown no dilatation proving that the female adult mosquitoes over winter as nuliparus and the last blood they took will be used for producing the fat body allowing the female to start the next generation in the beginning of next seasonal activity. However, it remains un-clear how these ecological data fit with the presence of one or more species within *An. maculipennis *complex in northern Iran, and what is the role of other anophelines in this region (i.e. *An. superpictus, An. hyrcanus, An. claviger, An. algeriensis, Anopheles pseudopictus*) in malaria transmission and since re-introduction of malaria in northern Iran.

## Conclusion

Beklemishev [54] listed eight major factors that determine epidemiological efficacy of malaria vectors in Russia: susceptibility of mosquitoes to Plasmodium parasites, sporozoite survival in salivary glands, female feeding behavior, absolute and relative number of mosquitoes, seasonal dynamics of mosquito densities, survival rate and infective period of mosquito females, ambient temperature and winter diapauses of adult females in a state of gonotrophic dissociation. Besides previous studies indicated that the sibling species are not equally important as vectors for malaria parasites because of their feeding preferences and their differential susceptibility to infection, as described in complex species of *Anopheles culicifacies*, *Anopheles gambiae*, *Anopheles fluviatilis *[10,55-59]. This may be grounds for re-considering the importance of other previously identified species in this region and their role in malaria transmission.

Nowadays, the most important vectors are considered to be *An. sacharovi*, which is responsible for the majority of *Plasmodium vivax *transmissions in the Asian part of Turkey [60], and *An. labranchiae*, formerly was the main vector of malaria in Italy [9]. *An. atroparvus *is the most efficient vector in Britain [43]. *An. maculipennis*, *An. messeae *and *An. sacharovi *are capable of transmitting malaria; however, they exhibit different vector capacities [8,60,61]. In this regard, detection of three species of *An. maculipennis *complex including *An. atroparvus, An. messae *and *An. labranchiae*, as new records in northern Iran, is a case for concern because of their potential in malaria transmission and more important, the extent of their geographical distribution towards southern territory of the *An. maculipennis *complex.

A better understanding on the epidemiology of malaria on both sides of the Caspian Sea may be provided by applying the molecular techniques to enable the correct identification of species complexes, the detection of plasmodium composition in anopheles vectors and the status of insecticide resistance by looking at related genes. In addition, it is worth remembering that as the *An. maculipennis *complex is distributed over an area that covers central Iran to the Caspian Sea region and up to Sweden, an international effort is required to prevent the re-introduction of malaria to those areas where "anophelism without malaria" prevails.
